# Immunohistochemical Expression of CK13 and Molecular Analysis of *KRT13* and *APC* in Odontogenic Ghost Cell Lesions, Adenoid Ameloblastoma, and Conventional Ameloblastoma

**DOI:** 10.1007/s12105-026-01938-8

**Published:** 2026-06-24

**Authors:** Lucas Fabian Polti, Juan Manuel Arteaga Legarrea, Estefanía Sicco, Felipe Martins Silveira, Lauren Frenzel Schuch, Vanesa Pereira Prado, Ronell Bologna Molina, María Luisa Paparella, Fernanda Faria Rocha, Marina Gonçalves Diniz, Ricardo Santiago Gomez, Silvia Ferreira de Sousa, Felipe Paiva Fonseca

**Affiliations:** 1 Department of Oral Surgery and Pathology, School of Dentistry, Universidade Federal de Minas Gerais, Belo Horizonte, Brazil; 2 Department of Diagnosis in Pathology and Oral Medicine, Facultad de Odontología, Universidad de La República, Montevideo, Uruguay; 3 Unidad de Patología Quirúrgica, Facultad de Odontología, Universidad de Buenos Aires, Ciudad Autónoma de Buenos Aires, Argentina; 4 Department of Pathology, Biological Sciences Institute, Universidade Federal de Minas Gerais, Belo Horizonte, Brazil

**Keywords:** Calcifying odontogenic cyst, Dentinogenic ghost cell tumor, Adenoid ameloblastoma, Conventional ameloblastoma, *APC*, *KRT13*

## Abstract

**Purpose:**

The aim of this study was to evaluate the immunohistochemical expression of CK13 and specific mutations in *KRT13* and *APC* genes in cases of calcifying odontogenic cyst (COC), dentinogenic ghost cell tumor (DGCT), adenoid ameloblastoma (AA), and conventional ameloblastoma (CA).

**Materials and Methods:**

Twenty-nine cases (22 COC, 2 DGCT, 1 AA, and 4 CA) were collected from two diagnostic centers. Immunohistochemical analysis of CK13 expression and polymerase chain reaction (PCR)-based molecular investigation of specific mutations (*APC* E1080* and *KRT13* M239V and Y281H) were performed.

**Results:**

CK13 expression in COC was observed in the suprabasal/superficial layers of the cystic epithelium in 14 cases-64%; ghost cells showed positivity in 12 cases-54%. DGCT cases were negative in the epithelial proliferation but positive in ghost cells. The AA case was negative. Three CA cases demonstrated positivity in suprabasal/central cells. None of the cases harbored the investigated mutations. However, the intronic polymorphism *KRT13* c.735 + 10A > G (dbSNP rs7211235) was identified in 16 COC cases, one DGCT case, one AA case, and one CA case, whereas *KRT13* c.735 + 6C > T (dbSNP rs181122697) was detected in one COC case. Additionally, one case harbored a previously unreported silent/synonymous mutation, *KRT13* c.690G > A (p.E230E), of unknown significance.

**Conclusions:**

CK13 expression in COC and CA suggests squamous differentiation of odontogenic epithelium. The detected *KRT13* genetic variations are probably not associated with tumorigenic mechanisms in COC, DGCT, AA, and CA. The *APC* E1080* mutation was not identified in any of the entities included in the present study. Further studies are therefore required to more precisely define the genetic profile of these entities and, particularly, to clarify the potential biological relationship between dentinogenic ghost cell tumor and adenoid ameloblastoma.

## Introduction

The most recent classification of odontogenic cysts and tumors proposed by the World Health Organization (WHO, 5th edition, 2022) recognizes the calcifying odontogenic cyst (COC), dentinogenic ghost cell tumor (DGCT), and odontogenic ghost cell carcinoma (OGCC) as the pathological entities comprising the group of odontogenic ghost cell lesions (OGCL) [1]. Additionally, in this classification, adenoid ameloblastoma (AA) was recognized and incorporated as a new, distinct clinicopathologic entity. The essential morphologic diagnostic criteria include ameloblastoma-like proliferation, cribriform architecture, duct-like structures, and whorled or morule-like cellular condensations. Dentinoid deposition, clear cells, and focal accumulation of ghost cells are considered desirable criteria [1]. Therefore, this entity exhibits an overlap or combination of morphologic patterns recognized in other odontogenic disorders, such as conventional ameloblastoma (CA), adenomatoid odontogenic tumor (AOT), and OGCL, and may conceptually be interpreted as a hybrid odontogenic lesion [2, 3]. Furthermore, essential diagnostic criteria of AA have been identified in cases of COC [4–7] and particularly in DGCT [7–10], raising questions as to whether these lesions should be interpreted as independent entities [11–13] or as manifestations within a single spectrum [4–10].

The implementation of immunohistochemical and, particularly, molecular studies has attempted to clarify these issues. Current evidence indicates that mutations affecting the *BRAF* and *KRAS* genes constitute distinctive genetic alterations in CA and AOT, respectively, allowing their molecular distinction from AA [14, 15]. However, AA has demonstrated involvement of the WNT/β-catenin pathway, with nuclear accumulation of this protein identified by immunohistochemistry, as well as mutations in its encoding gene, *CTNNB1* [9, 11], indicating an immunohistochemical and genetic profile like that described in OGCL [4, 8, 16]. Moreover, cases of AA and OGCL showing β-catenin immunoexpression in the absence of mutations in the corresponding gene have been reported, in which alterations affecting other components of the WNT pathway were identified, including tumor suppressor genes such as adenomatous polyposis coli (*APC*) and E3 ubiquitin ligases, smad ubiquitination regulatory factor 1 (*SMURF1*) and neural precursor cell expressed, developmentally down regulated 4-like (*NEDD4L*) [4, 8]. In addition, isolated cases of DGCT and AA have demonstrated *KRT13* gene alterations associated with the possible mechanism of ghost cell formation, considering that these cells are regarded as the result of an aberrant keratinization process [8], although the biological impact of the mutations remains questionable [17].

At the epidermal level, WNT signaling plays a fundamental role in regulating the balance between basal cell self-renewal and cellular differentiation, contributing to the maintenance of tissue homeostasis. Moreover, in epithelial differentiation processes, particularly within the hair follicle, the WNT pathway has been shown to indirectly modulate the expression of specific keratins through intermediary transcription factors, suggesting its involvement in cellular differentiation and keratinization mechanisms [18]. In this context, it is plausible that alterations in the WNT/β-catenin pathway may influence cytokeratin expression in cystic and neoplastic odontogenic lesions, particularly those exhibiting ghost cells.

At the immunohistochemical level, CK13 expression has been associated with epithelial differentiation and maturation, typically showing positivity in the suprabasal layers of stratified squamous epithelium. Expression of this cytokeratin has also been reported in certain odontogenic entities, including COC and CA, supporting the occurrence of squamous differentiation phenomena during the progression of these lesions [19–21].

Regarding ghost cells, recent immunohistochemical studies suggest that these epithelial cells may represent the result of a degenerative process associated with lysosomal increase and protein accumulation (keratins, amelogenins) [7].

Considering these findings, the tumorigenic molecular events involved in the development of OGCL and AA appear to be closely related to the participation of different components of the WNT/β-catenin pathway. Therefore, further investigation is required to better understand the biological profile of these lesions and to achieve consensus regarding their nomenclature and classification. Although CA is not typically associated with recurrent WNT/β-catenin pathway alterations or the presence of ghost cells, its morphological features may frequently be identified in OGCL and AA, constituting an important comparative parameter in immunohistochemical and molecular studies.

The aim of this study was to evaluate the immunohistochemical expression of CK13 and specific mutations in the *KRT13* and *APC* genes in cases of calcifying odontogenic cyst, dentinogenic ghost cell tumor, adenoid ameloblastoma, and conventional ameloblastoma.

## Materials and Methods

A total of 29 cases (22 calcifying odontogenic cysts, 2 dentinogenic ghost cell tumors, 1 adenoid ameloblastoma, and 4 conventional ameloblastomas) were retrieved from the Unidad de Patología Quirúrgica, Facultad de Odontología, Universidad de Buenos Aires, Argentina, and from the Department of Oral Surgery and Pathology, School of Dentistry, Universidade Federal de Minas Gerais, Brazil. This study was approved by the Research Ethics Committee of the Universidade Federal de Minas Gerais, Brazil (protocol number: 89170725.5.0000.5149) and by the Ethics Committee of the Facultad de Odontología, Universidad de Buenos Aires (Nr006/2019).

Clinical and radiographic information was obtained from archival records. Histopathological features were reviewed and confirmed by two oral pathologists. Only cases not subjected to decalcification during histological processing and with sufficient material available in paraffin blocks for immunohistochemical and molecular analyses were included. Seventeen of the 22 COC cases included in this study were part of a previously published series of 69 odontogenic ghost cell lesions, in which the immunohistochemical profile was evaluated using an extensive antibody panel and the molecular profile was investigated to identify potential mutations in the *CTNNB1* gene [7]. One DGCT case included in the present study had been previously reported as a case report [22].

### Immunohistochemical Analysis

For immunohistochemical processing, 3-µm-thick tissue sections were subjected to antigen retrieval using a pressure cooker and retrieval solution (citrate buffer, pH 6.0) for 30 min, followed by blocking of endogenous peroxidase activity with hydrogen peroxide baths for 15 min. Subsequently, the slides were incubated with the primary antibody anti-cytokeratin 13 (CK13; clone KS-1A3; Invitrogen; USA; dilution 1:50) for one hour. Samples were incubated with the rabbit linker and thereafter with the polymer, each for 30 min. Immunoreactions were visualized using diaminobenzidine (DAB), and counterstaining was performed with Mayer’s hematoxylin. Immunohistochemical staining was performed using the EnVision FLEX Detection System (Dako, Agilent Technologies, Glostrup, Denmark). Prior to immunohistochemical evaluation, the criteria for the interpretation of CK13 expression were established by consensus between the first and last authors. Subsequently, given the qualitative nature of the immunohistochemical assessment, all cases were evaluated using a consensus-based approach. A fragment of lining oral mucosa was used as an external positive control. CK13 expression was evaluated in the epithelial lining in cystic cases and in the epithelial proliferation in neoplastic cases, considering basal layer staining separately from suprabasal and superficial layers. In COC cases, expression was also compared between ameloblastic and ameloblastomatous regions. In the present study, the term “ameloblastic epithelium” was used to describe odontogenic epithelium with an embryonic appearance, showing differentiation similar to the reduced enamel epithelium, without frankly neoplastic histological features. In contrast, “ameloblastomatous epithelium” was used to refer to epithelium exhibiting morphological features similar to those observed in ameloblastoma, including hyperchromatic cuboidal or columnar basal cells with reverse nuclear polarity and subnuclear vacuolization resembling ameloblasts, as well as suprabasal layers similar to the stellate reticulum of the enamel organ, regardless of whether these features were present in conventional ameloblastomas or in other odontogenic lesions [23]. Expression was classified as focal when positivity was observed in less than 50% of cells and as diffuse when positivity exceeded 50%. In addition, staining intensity was assessed and categorized as low ( +), moderate (+ +), or strong (+ + +). The same parameters of focality/diffuseness and intensity were applied to ghost cells. The entirety of the histological section in each case was analyzed using different microscopic magnifications (40 × , 100 × , 200 × , and 400 ×).

### Molecular Analysis

Molecular analysis included assessment of specific alterations in the *APC* and *KRT13* genes: the nonsense mutation *APC* E1080* and the missense mutations *KRT13* M239V and *KRT13* Y281H, previously reported [8]. DNA extraction was performed using the QIAamp DNA FFPE Tissue Kit (QIAGEN, Hilden, Germany), according to the manufacturer’s instructions. DNA concentration and quality were assessed using a NanoDrop ND-2000c spectrophotometer (Thermo Fisher Scientific, Wilmington, DE, USA). Primer sequences were designed using the freely available NCBI Primer-BLAST application and included: *APC* E1080*, forward, 5’-CAGAATGAAAGATGGGCAAGAC-3’ and reverse, 5’-CCATGATTAGAACCCACTCGAT-3’(227 bp); *KRT13* M239V, forward, 5’-TATGAGAATGAGCTGGCCCTG-3’ and reverse, 5’-TCTCACTGGAGGTTGTTGAGC-3’ (204 bp); and *KRT13* Y281H, forward, 5’-CAGGTCAACGTGGAGATGGA-3’ and reverse, 5’-CTGGCTATATGGGATGGGCT-3’ (173 bp). Gene amplification was performed using the BioMixCell Flash HS PCR MasterMix Kit (Biocell Biotecnologia LTDA, Belo Horizonte, Brazil) with 12.5 µL of BioMix and 1 µL of each forward and reverse primer per sample. Conventional PCR consisted of an initial denaturation at 95 °C for 3 min, followed by 45 three-step cycles: 15 s at 95 °C (denaturation), 15 s of annealing at the following temperatures: *APC* E1080* (55 °C), *KRT13* M239V (57 °C), and *KRT13* Y281H (57 °C), and 30 s at 72 °C (extension). A final extension step was performed at 72 °C for 10 min. PCR products were verified by electrophoresis on 3% agarose gel using TAE 1X running buffer at a constant voltage (200 V). PCR product purification was carried out using ExoSAP-IT enzyme (Affymetrix, USB®, Santa Clara, CA, USA) according to the manufacturer’s instructions. Sanger sequencing was performed at the Rede de Plataformas Tecnológicas, Fundação Oswaldo Cruz (FIOCRUZ), Brazil. Sequences were aligned and analyzed using SnapGene® Viewer version 4.3.4 software.

## Results

### Clinical, Radiographic, and Histopathological Features

Table 1 summarizes the clinical, immunohistochemical, and molecular features of the cases included in this study.

**Table 1:** Summary of the clinical, immunohistochemical, and molecular features of the COC, DGCT, AA, and CA cases

Case	Sex/age	Location	Immunohistochemistry: CK13			Molecular analysis			
			BC	SBC	GC	*APC* E1080*	*KRT13* M239V	*KRT13* Y281H	Other findings
*COC*									
1*	F/45	Mn (ant)	Neg	Focal ( +)	Neg	WT	WT	WT	-
2	M/58	Left mx (ant-post)	Neg	Focal (+ + +)	Neg	WT	WT	NA	*KRT13*: c.735 + 10 A > G (i)
3	M/7	Right mn (post)	Neg	Focal ( +)	Focal ( +)	WT	WT	WT	*KRT13:* c.735 + 10 A > G (i) *KRT13:* c.690 G > A /p.E230E (e)
4	F/13	Mx	Neg	Focal (+ +)	Neg	WT	WT	NA	*KRT13*: c.735 + 10 A > G (i)
5	M/14	Mx (ant)	Focal (+ + +)	Focal (+ + +)	Focal (+ + +)	WT	WT	NA	-
6	F/64	Mx (ant)	Neg	Neg	Focal ( +)	WT	WT	WT	*KRT13*: c.735 + 10 A > G (i)
7	M/24	Mn (ant)	Neg	Neg	Neg	WT	WT	WT	-
8	F/77	Mx (ant)	Neg	Neg	Neg	WT	WT	WT	*KRT13*: c.735 + 10 A > G (i)
9	M/13	Mn (ant)	Neg	Focal ( +)	Neg	WT	WT	WT	*KRT13:* c.735 + 10 A > G (i)
10	F/19	Left mx (ant-post)	Neg	Focal ( +)	Focal ( +)	WT	WT	WT	*KRT13:* c.735 + 10 A > G (i)*KRT13*: c.735 + 6 C > T (i)
11	F/26	Mn (ant)	Neg	Neg	Focal ( +)	WT	WT	WT	*KRT13*: c.735 + 10 A > G (i)
12	M/49	Mn (ant)	Neg	Focal ( +)	Focal ( +)	WT	WT	WT	*KRT13*: c.735 + 10 A > G (i)
13	F/52	Left mn (post)	Neg	Focal (+ +)	Focal (+ +)	WT	WT	NA	*KRT13*: c.735 + 10 A > G (i)
14	M/64	Mn (ant)	Neg	Neg	Neg	WT	WT	WT	*KRT13:* c.735 + 10 A > G (i)
15	M/36	Left mn (post)	Focal (+ +)	Focal (+ +)	Focal (+ +)	WT	WT	WT	-
16	M/56	Mx (ant)	Neg	Focal (+ +)	Neg	WT	WT	WT	*KRT13:* c.735 + 10 A > G (i)
17	F/15	Right mn (post)	Neg	Focal (+ +)	Focal (+ +)	WT	WT	WT	*KRT13:* c.735 + 10 A > G (i)
18	F/26	Mx (ant)	Neg	Neg	Neg	WT	WT	WT	*KRT13:* c.735 + 10 A > G (i)
19	M/27	Left mx (post)	Neg	Neg	Focal ( +)	WT	WT	WT	*KRT13:* c.735 + 10 A > G (i)
20	F/13	Mx	Focal (+ +)	Focal (+ +)	Focal (+ +)	WT	WT	WT	*KRT13:* c.735 + 10 A > G (i)
21	M/63	Mn (ant)	Neg	Focal (+ + +)	Neg	WT	NA	WT	-
22	F/45	Mx (ant)	Neg	Neg	Focal (+ + +)	WT	NA	WT	-
*DGCT*									
23	F/50	Mx	Neg	Neg	Focal ( +)	WT	WT	WT	-
24	F/60	Right mx (post)	Neg	Neg	Focal ( +)	WT	WT	WT	*KRT13:* c.735 + 10 A > G (i)
*AA*									
25	37/F	Left mn (post)	Neg	Neg	Absent	WT	WT	WT	*KRT13*: c.735 + 10 A > G (i)
*CA*									
26	M/81	Left mx (post)	Neg	Focal (+ + +)	-	WT	WT	WT	*KRT13:* c.735 + 10 A > G (i)
27	M/54	Right mx (post)	Neg	Focal ( +)	-	WT	NA	NA	-
28	M/54	Left mn (post)	Neg	Neg	-	WT	WT	NA	-
29	M/33	Right mn (ant-post)	Neg	Focal (+ +)	-	WT	NA	WT	-

COC: calcifying odontogenic cyst. DGCT: dentinogenic ghost cell tumor. AA: adenoid ameloblastoma. CA: conventional ameloblastoma. 1*: peripheral case. BC: basal cells. SBC: suprabasal cells. GC: ghost cells. F: female. M: male. Mn: mandible. Mx: maxilla. Ant: anterior. Post: posterior. Neg: negative. WT: wild type. NA: not available. (i): intronic. (e): exonic. ( +): low expression. (+ +): moderate expression. (+ + +): intense expression

**Fig. 1:**
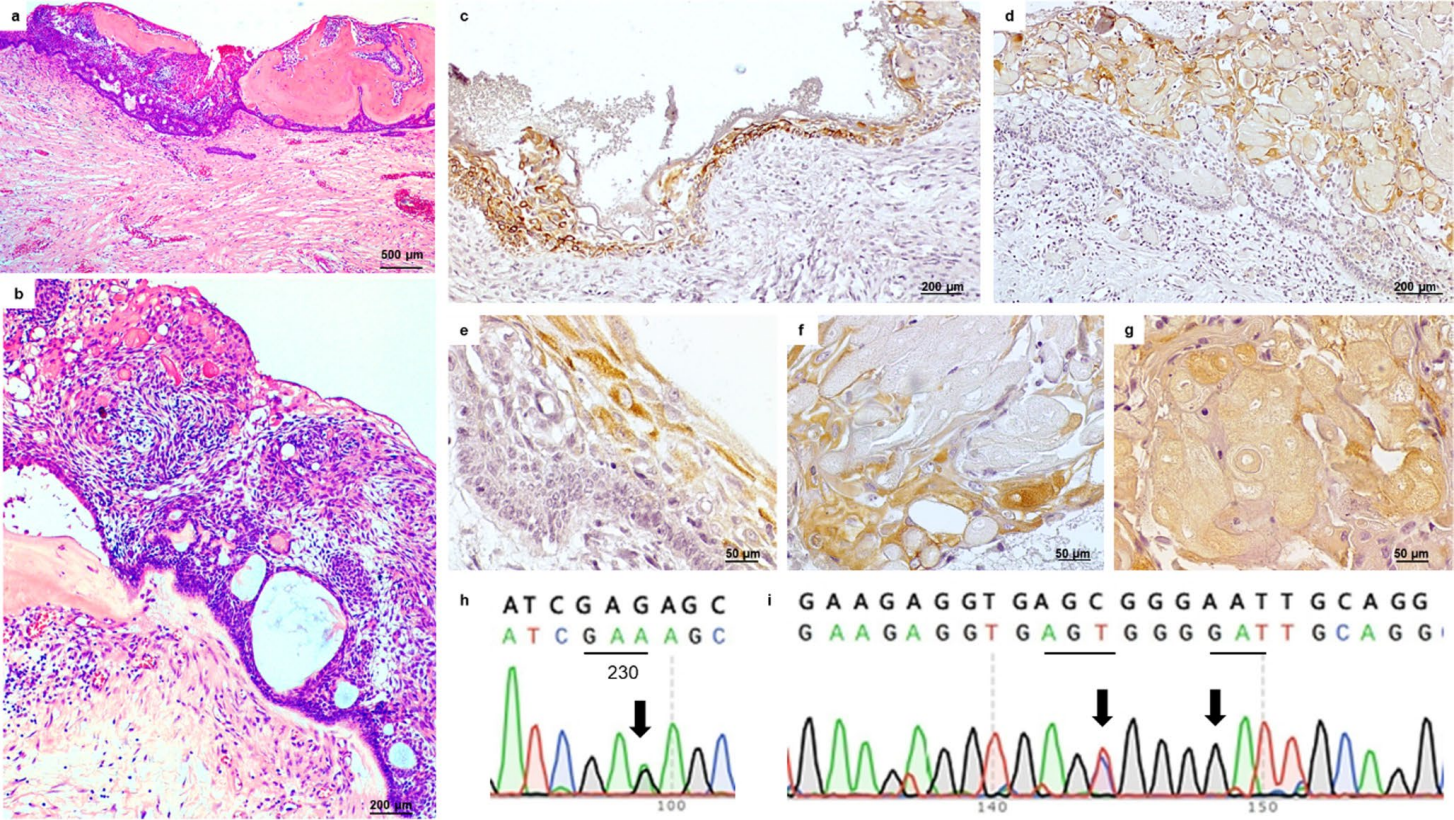
COC. **a** Cystic wall lined by ameloblastomatous epithelium exhibiting clusters of ghost cells and juxtaepithelial dentinoid deposition (H&E, Orig. Mag. 40 ×). **b** Ameloblastomatous epithelial lining with focal presence of ghost cells, pseudo-ductal structures, whorled cellular condensations, and juxtaepithelial dentinoid (H&E, Orig. Mag. 100 ×). **c**–**g** Immunohistochemical reaction for CK13. **c** Ameloblastic lining showing positivity in basal and suprabasal cells (Orig. Mag. 100 ×). **d** Negative ameloblastomatous lining with positive staining in ghost cells (Orig. Mag. 100 ×). **e** Ameloblastomatous epithelium exhibiting positivity restricted to superficial layers (Orig. Mag. 400 ×). **f**–**g** Focal positivity in ghost cells (Orig. Mag. 400 ×). **h**–**i** *KRT13* forward chromatogram. h) A heterozygous position is observed at nucleotide G > A at codon 230 (Case 3). i) C > T and A > G substitutions in the intronic region between exons 3 and 4 (Case 10)

Twenty-two cases of COC were included. Sex distribution was equal, with a wide age range (7–77 years) and a mean age of 37 years. Lesions were equally distributed between the maxilla and mandible, with anterior predilection in 13 cases (59%). Twenty-one cases (95%) were intraosseous (central), whereas one case was extraosseous (peripheral) (5%). Imaging studies, available in eight cases (36%), predominantly demonstrated unilocular radiolucent osteolytic lesions in seven cases (87%) and, in one case (13%), a radiolucent lesion with radiopaque foci. Microscopically, all cases exhibited cystic architecture, with an epithelial lining predominantly composed of ameloblastomatous areas. Ghost cells were identified in all analyzed cases. Additionally, focal morule-like cellular condensations were observed in 10 cases (45%), pseudo-ductal structures lined by a single layer of cuboidal cells in seven cases (32%), and a cribriform pattern in one case (4%). In the underlying connective tissue, dentinoid material deposition was identified in 12 cases (54%), and a giant cell reaction associated with ghost cells was observed in eight cases (36%) (Fig. 1a, b).

**Fig. 2:**
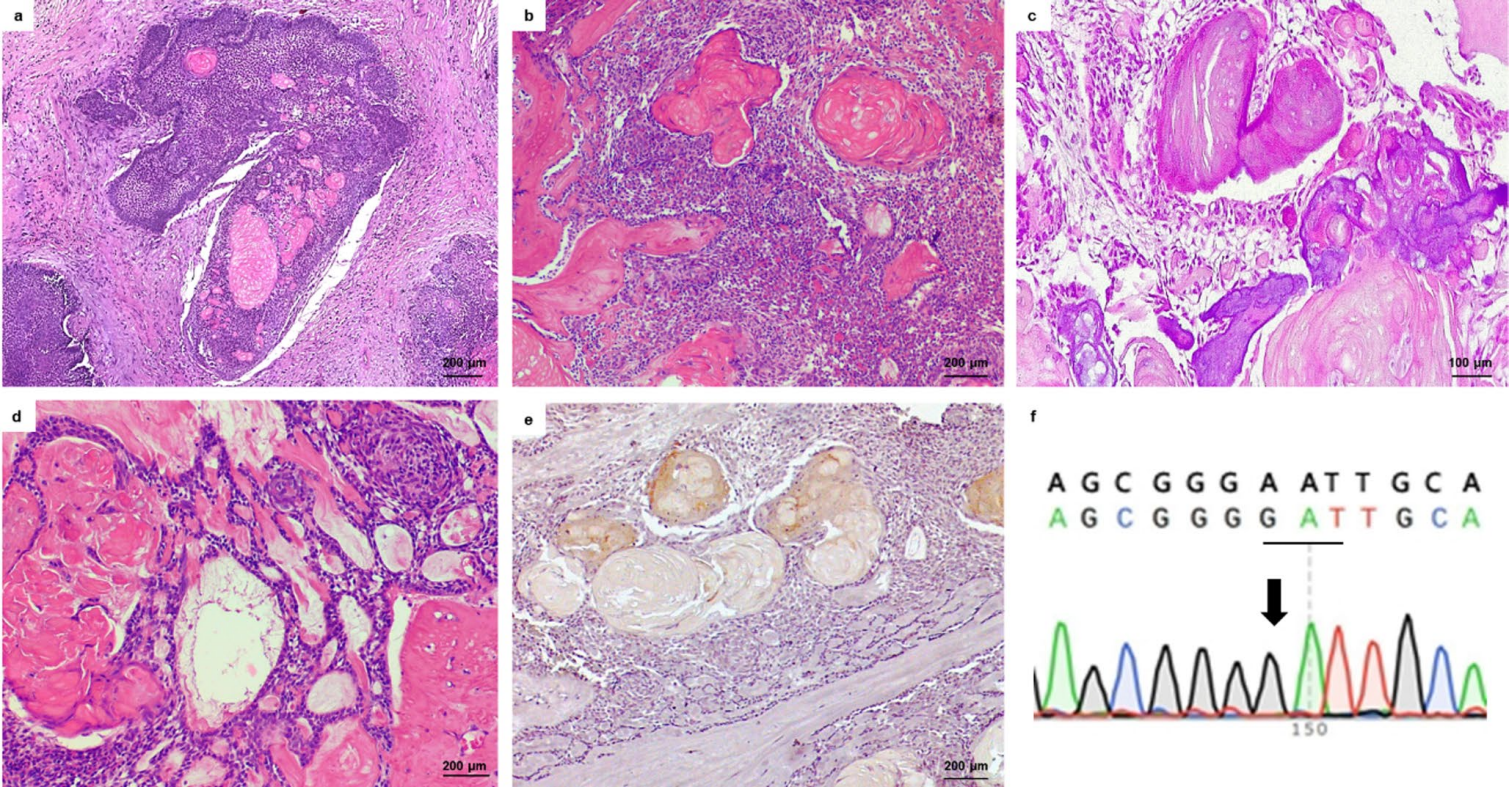
DGCT. **a**–**b** Epithelial proliferation exhibiting ghost cells and dentinoid material (H&E, Orig. Mag. 100 ×). **c** Clusters of ghost cells showing focal calcification (H&E, Orig. Mag. 200 ×). **d** Area displaying pseudo-ductal structures, morule-like cellular condensation, dentinoid deposition, and ghost cells (H&E, Orig. Mag. 100 ×). **e** Immunohistochemistry for CK13 showing focal positivity in ghost cells and negative staining in the epithelial proliferation (Orig. Mag. 100 ×). **f** *KRT13* forward chromatogram showing an A > G substitution in the intronic region between exons 3 and 4 (Case 24)

Two DGCT cases were analyzed. Both occurred in women aged 50 and 60 years, presenting lesions in the maxilla, one without precise anatomical specification and the other located in the right posterior region. Both cases were intraosseous, also involving oral mucosa and soft tissues. Tomographic imaging available for the second case demonstrated a poorly defined hypodense lesion with hyperdense foci involving the right maxillary sinus, nasal cavity, and pterygoid process. Microscopically, both cases showed ameloblastomatous proliferation with abundant ghost cells, with focal calcification identified in one case. The epithelial proliferation exhibited morule-like areas in both cases and pseudo-ductal structures in one case. Dentinoid material was identified in the stroma of both lesions (Fig. 2a–d).

**Fig. 3:**
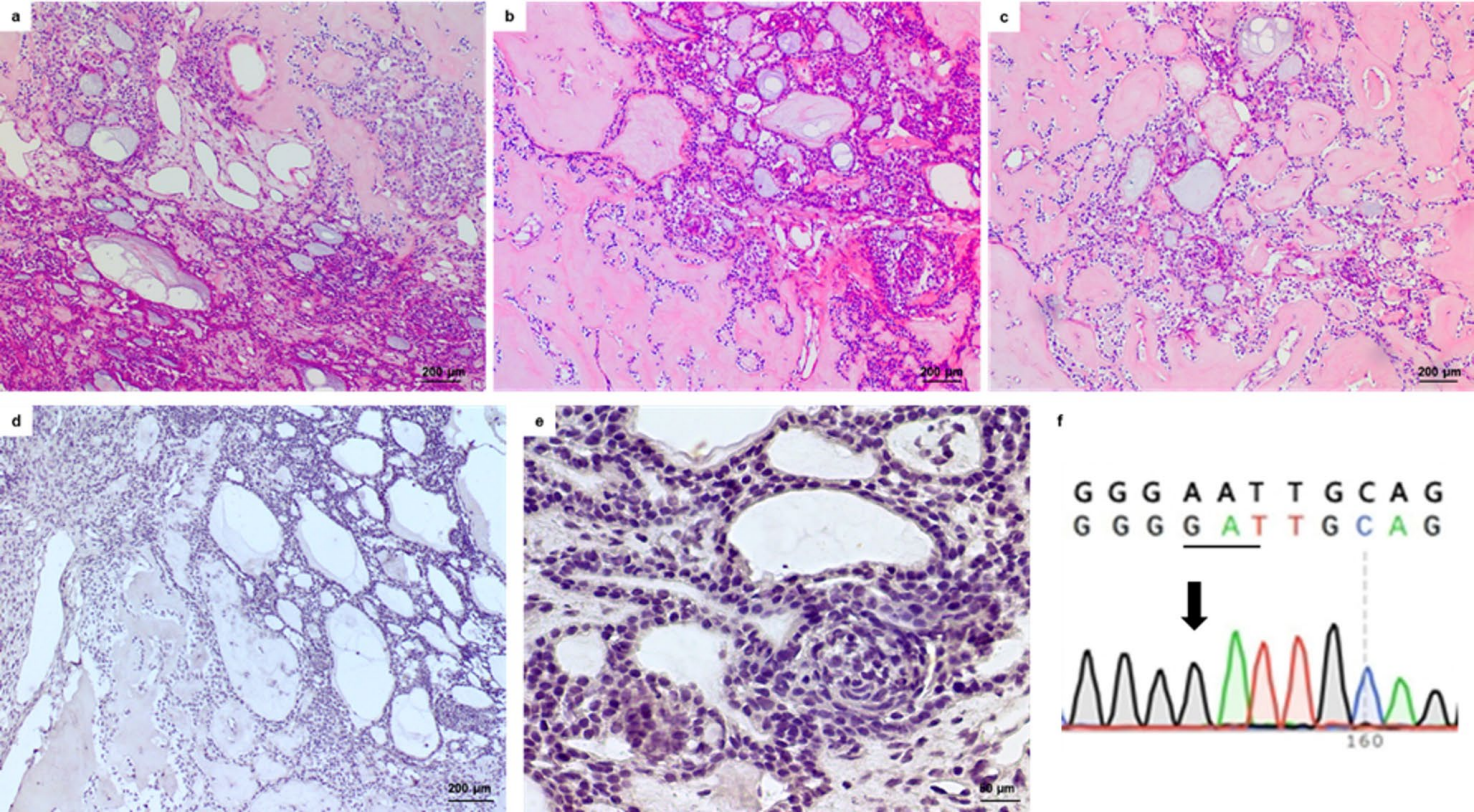
AA. **a**–**c** Epithelial proliferation exhibiting a cribriform pattern, pseudo-ductal structures, occasional morule-like cellular condensations and abundant dentinoid material with entrapment of clear cells (H&E, Orig. Mag. 100 ×). **d**–**e** Immunohistochemistry for CK13 showing absence of expression in the epithelial proliferation (d–Orig. Mag. 100 × ; e–Orig. Mag. 400 ×). **f** *KRT13* forward chromatogram showing an A > G substitution in the intronic region between exons 3 and 4 (Case 25)

The single AA case included in this study corresponded to a 37-year-old woman presenting a unilocular radiolucent lesion in the mandible, located in the left premolar region. Microscopic examination revealed epithelial proliferation with predominantly cribriform architecture, composed of ameloblast-like basal cells showing reverse nuclear polarity and a minor suprabasal component resembling stellate reticulum. Duct-like structures formed by cuboidal to columnar cells were also identified, along with focal whorled cellular condensations reminiscent of morules. Abundant dentinoid-like mineralized material with entrapment of odontogenic epithelial cords composed of clear cytoplasmic cells was widely observed. Ghost cells were not identified (Fig. 3a–c).

**Fig. 4:**
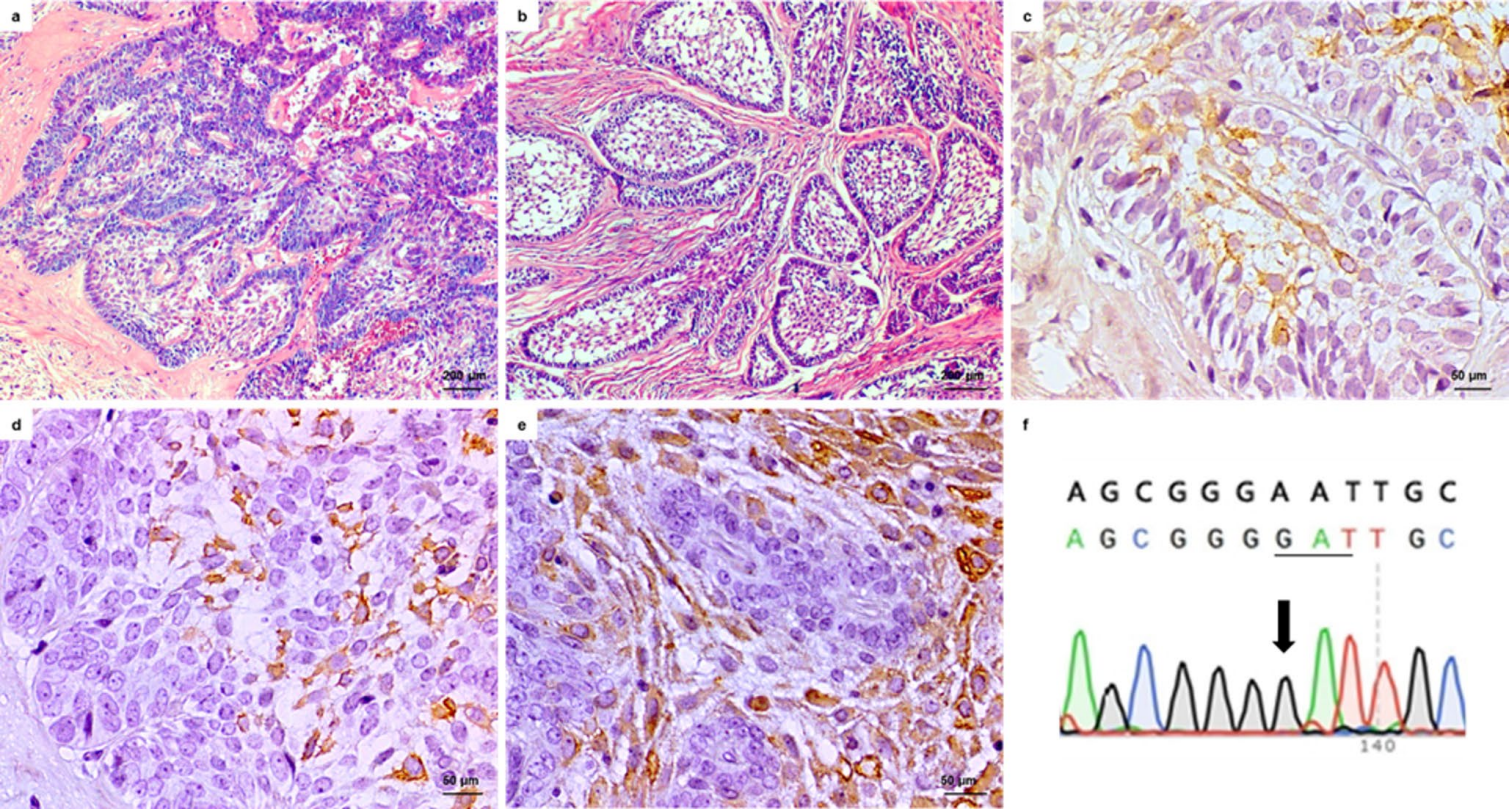
CA. **a** Ameloblastomatous proliferation exhibiting a follicular-plexiform pattern (H&E, Orig. Mag. 100 ×). **b** Ameloblastomatous epithelium with a follicular pattern (H&E, Orig. Mag. 100 ×). **c**–**e** Immunohistochemistry for CK13 showing positivity in central spindle-shaped cells resembling the stellate reticulum of the enamel organ and negativity in peripheral-basal cells (Orig. Mag. 400 ×). **f** *KRT13* forward chromatogram showing an A > G substitution in the intronic region between exons 3 and 4 (Case 26)

Regarding CA, four cases were included. All occurred in men, with ages ranging from 33 to 81 years (mean age: 55 years). Two cases were located in the mandible (one anterior and one anteroposterior), whereas the remaining two were located in the maxilla, both in the posterior region (one right and one left). All cases were intraosseous (central). Radiographic information was available for two cases, describing multilocular radiolucent osteolytic lesions. Microscopically, all cases demonstrated ameloblastomatous proliferation, exhibiting a follicular pattern in three cases and a follicular-plexiform pattern in one case (Fig. 4a–b).

### Immunohistochemical Results

CK13 expression in COC cases was focally observed in the suprabasal and superficial layers of the cystic epithelial lining in 14 cases (64%), showing variable staining intensities: weak (5 cases, 36%), moderate (6 cases, 43%), and strong (3 cases, 21%). Basal layer staining was identified in only three cases (14%). Furthermore, CK13 positivity within the epithelial lining was predominantly observed in ameloblastic epithelial areas, characterized by a reduced number of cell layers (9 cases, 64%), whereas expression in ameloblastomatous epithelium was identified in five cases (36%). Ghost cells exhibited focal positivity in 12 cases (54%), with heterogeneous staining intensities: weak (6 cases, 50%), moderate (4 cases, 33%), and strong (2 cases, 17%). Four cases (18%) were completely negative (Fig. 1c–g). Both DGCT cases showed a similar expression pattern, with negativity in the epithelial proliferation and focal weak staining in ghost cells (Fig. 2e). The AA case was negative (Fig. 3d–e). Among the four CA cases, all were negative in peripheral basal cells, whereas suprabasal-central cells exhibited focal positivity in three cases, with one case each showing weak, moderate, and strong staining intensity, respectively (Fig. 4c–e).

### Molecular Findings

Sequence analysis of the 29 included cases revealed no evidence of *APC* E1080*, *KRT13* M239V, or *KRT13* Y281H mutations.

However, the following single nucleotide variants (SNVs) were identified: the intronic variant *KRT13* c.735 + 10 A > G, located between exons 3 and 4, which was identified in 16 COC cases (Fig. 1i) in one DGCT (Fig. 2f), one AA (Fig. 3f), and one CA (Fig. 4f). Additionally, COC case no. 3 exhibited the silent/synonymous mutation *KRT13* c.690 G > A (p.E230E), whereas case no. 10 showed the intronic variant KRT13 c.735 + 6 C > T (Fig. 1h–i).

## Discussion

The findings of the present study demonstrated focal and heterogeneous CK13 expression in COC cases, with positivity also detected in ghost cells in more than half of the cases, whereas expression was limited in DGCT, absent in AA, and focal in CA. At the molecular level, previously reported mutations in *APC* E1080* and *KRT13* M239V and Y281H [8] were not identified; instead, intronic variants classified as benign germline polymorphisms and one silent/synonymous mutation were detected in *KRT13* gene. Collectively, these results suggest that *KRT13* does not play a determinant role in the tumorigenesis of these entities or in ghost cell formation and reinforce the need to investigate other components of the WNT/β-catenin pathway to better understand the biological relationship between OGCL and AA. Although CA is not typically associated with ghost cell formation or recurrent WNT/β-catenin pathway alterations, its inclusion in the present study allowed the establishment of a comparative model, given that it shares morphological features with OGCL and AA.

Several authors have highlighted significant similarities between AA and DGCT, raising ongoing debates regarding their nosological boundaries. Notably, morphological patterns considered essential for AA diagnosis have also been identified in OGCL, particularly in DGCT [4–10]. Therefore, establishing clear and reproducible diagnostic criteria capable of accurately distinguishing cribriform patterns from duct-like structures, differentiating epithelial whorls from true morules, and defining an acceptable quantitative threshold of ghost cells in AA would substantially improve the microscopic diagnosis of these lesions [24]. However, not all cases diagnosed as AA demonstrate ghost cells [25], which explains why they are not considered an essential diagnostic criterion, introducing additional interpretative complexity. Consequently, debate persists regarding whether these lesions should remain independent pathological entities [11–13] or instead be regarded as part of a single nosological spectrum under the proposed designation “WNT Pathway-Altered Benign Odontogenic Tumors” [8]. Nevertheless, although nuclear and cytoplasmic β-catenin immunoexpression has been demonstrated in DGCT, this positivity has not been consistently correlated with hotspot mutations in *CTNNB1* [8, 9], and current evidence would be insufficient to establish a unified molecular classification [13, 17]. In the context of AA, a greater number of cases have demonstrated mutations in the *CTNNB1* gene [11, 13]. However, at least two cases of DGCT with malignant transformation, in which the coexistence of benign and malignant components was demonstrated, have reported mutations in the *CTNNB1* hotspot [26, 27]. This evidence, together with the findings of a recent epigenetic study showing that AA and DGCT cases form a molecularly distinct group compared to other odontogenic tumors, since no differentially methylated regions were identified between these entities, supports the possibility that OGCL and AA belong to the same pathogenic spectrum [28]. Further studies are therefore required to more precisely define the genetic profile of these entities. At present, cases exhibiting AA-like morphology in the presence of abundant ghost cells may be better interpreted as DGCT. Accordingly, such cases have been described as “AA-type DGCT” [10] or as “mixed tumors” [8]. It has also been suggested that only cases of AA showing the concomitant presence of ghost cells and dentinoid material, which are essential diagnostic criteria for DGCT, should be diagnosed as DGCT [29].

Literature reviews focusing on molecular alterations associated with odontogenic cysts and tumors have emphasized the significant involvement of the WNT/β-catenin pathway in tumorigenic processes, particularly in OGCL and AA [30–32]. Within this framework, mutations in the hotspot region of exon 3 of *CTNNB1*, especially at codons 32, 33, 34, 37, and 41, have been most frequently reported in COC [4, 33–35]. Less commonly, variants affecting codons 3, 4, 5, and 57 have also been described [36]. Additionally, isolated mutations have been identified in other WNT-related genes, including *APC* (Pro1433Leu; D170fs; E1327fs; E2003fs) and *NEDD4L* (E849fs) [4, 35]. Three cases harboring *DDR2* mutations of uncertain significance and one case presenting a *KRAS* activating mutation concomitant with *CTNNB1* mutation have also been reported [37]. Seventeen of the 22 COC cases included in the present study had previously been analyzed for SNVs in the *CTNNB1* hotspot, with only a single variation identified at codon 38 [7].

Regarding DGCT, molecular evidence remains limited. To date, only one case harboring a *CTNNB1* mutation at codon 3 has been reported [16, 36], along with one case showing a *BRAF* V600E mutation [9] and another demonstrating alterations in *APC* E1080* and *KRT13* M239V [8]. Several DGCT cases analyzed molecularly have lacked *CTNNB1* mutations despite persistent nuclear β-catenin immunostaining, suggesting activation of the WNT pathway [8, 9].

Conversely, a greater number of AA cases harbor *CTNNB1* hotspot mutations, predominantly affecting codons 33, 34, and 37 [9, 11]. Variants involving codons 31 and 68 have also been identified, together with additional mutations in genes potentially involved in tumorigenesis, including *TP53* (p.Asp393Asn), *ERBB2* (p.Val782Ile), and *PIK3CA* (p.His1048Tyr), suggesting possible cooperative molecular events [13]. Isolated *APC* mutation (E789fs) and occasional *BRAF* (V600E) mutations have also been reported [8, 9].

Additionally, two cases described as “mixed” due to marked morphological overlap between AA and DGCT exhibited heterogeneous molecular profiles: one harboring *SMURF1* (L424fs), *TP53* (P278S), and *KRT13* (Y281H) mutations, and another presenting a *NEDD4L* (N846fs) mutation [8].

Previously reported *APC* E1080* and *KRT13* M239V and Y281H mutations [8] were not identified in any case of the present series. These *KRT13* mutations were initially proposed to be associated with ghost cell formation because they were identified in two cases exhibiting abundant ghost cells [8]. However, other authors have argued against this hypothesis, recognizing that these *KRT13* mutations have been identified as germline variants in East Asian populations, particularly in Korean patients [17]. Additionally, 19 cases in this study (16 COC, 1 DGCT, 1 AA, and 1 CA) exhibited the same intronic A > G substitution at chromosomal position 735 + 10 between exons 3 and 4 of *KRT13* (https://www.ncbi.nlm.nih.gov/clinvar/variation/323093/)(dbSNP rs7211235). Furthermore, an intronic C > T substitution at position 735 + 6 was identified in one COC case (https://www.ncbi.nlm.nih.gov/clinvar/variation/323094/)(dbSNP rs181122697). Both variants are classified as benign in clinical databases and represent germline polymorphisms without evidence of pathogenic significance. However, it cannot be confirmed that these variants are germline in our cases, as only cystic and tumoral lesional tissue was analyzed. Finally, a previously unreported silent/synonymous mutation of unknown significance detected at codon 230 (GAG > GAA), encoding glutamic acid, was identified in another COC case. In the context of this study, these variants were identified in a South American population and further support the hypothesis that *KRT13* probably does not play a role in ghost cell formation or in odontogenic lesions capable of exhibiting these cells. Consistently, whole-exome sequencing studies have not identified mutations in other keratin genes in AA and DGCT cases [8].

From an immunohistochemical perspective, the study of cytokeratin expression has been extensively investigated in odontogenic tissues, given the role of odontogenic epithelium in tooth development as well as in multiple odontogenic cysts and tumors. Keratins constitute a superfamily of intermediate filaments specific to epithelial cells, composed of at least 20 distinct polypeptides whose expression is closely related to epithelial type and differentiation status [19, 20]. CK13, specifically, is a marker of epithelial differentiation or maturation restricted to suprabasal layers of stratified squamous epithelium. Its expression has been identified focally in the dental lamina and occasionally in reduced enamel epithelium of tooth germs, while in ameloblastoma and ameloblastic fibroma it has been observed in suprabasal stellate reticulum-like cells [19]. Similarly, CK13 positivity has been reported in suprabasal and superficial layers of the cystic epithelium of COC and occasionally in ghost cells [20, 21], although completely negative cases have also been described [38], reflecting expression variability. In the present study, focal CK13 positivity with heterogeneous intensity was identified in 14 of 22 COC cases, involving suprabasal and superficial epithelial layers, and ghost cells were positive in 12 cases. Greater expression was observed in ameloblastic areas compared with ameloblastomatous regions, suggesting that squamous maturation may be more advanced in epithelial areas with an embryonic appearance than in ameloblastoma-like regions. The two DGCT cases demonstrated focal positivity restricted to ghost cells, whereas the AA case was negative. Previous studies have reported low CK10/13 expression in these entities [9]. However, the limited number of DGCT and AA cases included in this study, together with the small number of cases investigated for CK13 expression in the literature, restricts the possibility of establishing more consistent comparisons. Regarding CA, three of four cases exhibited positivity restricted to suprabasal stellate reticulum-like cells. Taken together, these findings suggest that cystic epithelium in COC and neoplastic epithelium in CA undergo squamous differentiation, which appears less evident in DGCT and AA.

In line with these observations, epithelial proliferation and differentiation processes, as well as tooth developmental mechanisms, are associated with the WNT/β-catenin pathway [32, 39]. In this context, the WNT pathway may modulate keratin gene expression through intermediary transcription factors. For example, constitutive β-catenin activation has been shown to induce *KRT16* and *KRT17* expression via Pitx2, identifying this transcription factor as an important downstream effector involved in WNT- mediated hair follicle differentiation processes [18]. Additionally, Pitx2 and β-catenin have been demonstrated to interact synergistically to promote gene expression during odontogenesis. The Pitx2:β-catenin regulatory pathway is involved in epithelial cell differentiation and in the conversion of mesenchymal cells into amelogenin-expressing epithelial cells through miR-200a (microRNA) [40]. These findings support the hypothesis that, in pathological odontogenic epithelia, particularly in OGCL, abnormal activation of the WNT/β-catenin pathway may promote ectopic epithelial differentiation. In this setting, odontogenic epithelium could partially lose its normal odontogenic differentiation program and instead favor aberrant intracellular accumulation of cytokeratins and potentially amelogenesis-related proteins, ultimately contributing to ghost cell formation [35, 41, 42]. Consequently, ghost cells may represent the morphological expression of a degenerative process associated with abnormal protein accumulation [7] and dysregulation of the WNT/β-catenin pathway. This hypothesis is further supported by previous immunohistochemical findings demonstrating expression of epithelial and amelogenesis-related markers, including AE1/AE3 and amelogenin, in ghost cells, as well as focal positivity for CD68 and lysozyme, suggesting lysosomal involvement potentially related to degenerative phenomena [7]. Nevertheless, the precise role of the WNT/β-catenin pathway in epithelial keratinization processes, as well as its relationship with specific keratin alterations potentially involved in ghost cell formation, remains unclear and requires further investigation.

The limitations of the present study include the small sample size, particularly regarding the neoplastic entities, which restricts the possibility of extrapolating or generalizing the identified findings. Furthermore, difficulties in obtaining complete clinical and radiographic data in some cases were encountered, since the cases were retrospectively collected. Additionally, due to the storage time of some archival samples, the possibility of DNA degradation exists, which may have influenced the quality and reliability of the molecular analyses performed.

## Conclusions

Cytokeratin 13 expression in calcifying odontogenic cyst and conventional ameloblastoma suggests squamous differentiation of odontogenic epithelium in these entities.

The genetic variations detected in the *KRT13* gene are probably not associated with tumorigenic mechanisms involved in the development of odontogenic ghost cell lesions, adenoid ameloblastoma, and conventional ameloblastoma.

The *APC* E1080* mutation was not identified in any of the entities included in the present study.

Further studies are therefore required to more precisely define the genetic profile of these entities and, particularly, to clarify the potential biological relationship between dentinogenic ghost cell tumor and adenoid ameloblastoma.

## Data Availability

No datasets were generated or analysed during the current study.

## Declarations

### Stats Declaration

No statistical analysis was performed in the preparation of this manuscript.
